# Continuous Renal Replacement Therapy in Critically Ill Children in the Pediatric Intensive Care Unit: A Retrospective Analysis of Real-Life Prescriptions, Complications, and Outcomes

**DOI:** 10.3389/fped.2021.696798

**Published:** 2021-06-14

**Authors:** Emanuele Buccione, Francesco Guzzi, Denise Colosimo, Brigida Tedesco, Stefano Romagnoli, Zaccaria Ricci, Manuela L'Erario, Gianluca Villa

**Affiliations:** ^1^Pediatric Intensive Care Unit, Meyer Children's University Hospital, Florence, Italy; ^2^Neonatal Intensive Care Unit, AUSL Pescara, Pescara, Italy; ^3^Nephrology and Dialysis Unit, Meyer Children's University Hospital, Florence, Italy; ^4^Department of Experimental and Clinical Biomedical Sciences “Mario Serio”, University of Florence, Florence, Italy; ^5^Section of Anesthesiology and Intensive Care, Department of Health Sciences, University of Florence, Florence, Italy; ^6^Department of Anesthesia and Intensive Care, AOU Careggi, Florence, Italy

**Keywords:** acute kidney injury, hemodialysis, artificial membranes, blood clotting, vascular catheters, chronic kidney disease

## Abstract

**Introduction:** Severe acute kidney injury is a common finding in the Pediatric Intensive Care Unit (PICU), however, Continuous Renal Replacement Therapy (CRRT) is rarely applied in this setting. This study aims to describe our experience in the rate of application of CRRT, patients' clinical characteristics at admission and CRRT initiation, CRRT prescription, predictors of circuit clotting, short- and long-term outcomes.

**Methods:** A 6-year single center retrospective study in a tertiary PICU.

**Results:** Twenty-eight critically ill patients aged 0 to 18 years received CRRT between January 2012 and December 2017 (1.4% of all patients admitted to PICU). Complete clinical and CRRT technical information were available for 23/28 patients for a total of 101 CRRT sessions. CRRT was started, on average, 40 h (20–160) after PICU admission, mostly because of fluid overload. Continuous veno-venous hemodiafiltration and systemic heparinization were applied in 83.2 and 71.3% of sessions, respectively. Fifty-nine sessions (58.4%) were complicated by circuit clotting. At multivariate Cox-regression analysis, vascular access caliber larger than 8 Fr [HR 0.37 (0.19–0.72), *p* = 0.004] and regional citrate anticoagulation strategy [HR 0.14 (0.03–0.60), *p* = 0.008] were independent protective factors for clotting. PICU mortality rate was 42.8%, and six survivors developed chronic kidney disease (CKD), within an average follow up of 3.5 years.

**Conclusions:** CRRT is uncommonly applied in our PICU, mostly within 2 days after admission and because of fluid overload. Larger vascular access and citrate anticoagulation are independent protective factors for circuit clotting. Patients' PICU mortality rate is high and survival often complicated by CKD development.

## Introduction

Acute kidney injury (AKI) is a common complication in the Pediatric Intensive Care Unit (PICU), involving approximately one third of critically ill neonates and children (1, 2). Although Continuous Renal Replacement Therapy (CRRT) is usually applied in critically ill adult patients with severe AKI and/or multiple organ dysfunction syndrome (MODS) in order to support kidney function (3–5), this technique is uncommonly used in the PICU (6). In a large multicenter observational study, only 1.5% of critically ill children underwent CRRT (2). A knowledge gap exists regarding clinical and technical peculiarities of CRRT in the pediatric population (7), and several researchers in the field encourage the sharing of experiences and clinical and technical data (8–11). Predictors for circuit clotting are mostly unknown and originate from large cohorts of adult patients (12, 13). Uncertainties also exist on long-term kidney and global outcomes of critically ill pediatric patients who underwent CRRT. Although CRRT has been recognized as a strong predictor of short-term mortality, particularly when associated with fluid overload (FO) and MODS (14–17), pediatric AKI patients undergoing CRRT often encounter delays in referral to the nephrology unit or are lost to long-term follow-up (10, 18).

In this single center retrospective study we observe a cohort of pediatric critically ill patients with the aim of describing: (1) the rate of application of CRRT; (2) the demographic and clinical characteristics at PICU admission of patients treated with CRRT; (3) timing-to-start, indication, and technical prescription for CRRT; (4) baseline predictors (at CRRT initiation) of premature circuit clotting; (5) short- and long-term outcomes of CRRT patients, both in terms of PICU survival and nephrology follow-up.

## Materials and Methods

### Data Collection and Definitions

In this single center, retrospective, observational study, we included all consecutive patients aged 0 to 18 years who received CRRT during their PICU stay at the Meyer Children's Hospital (Florence, IT) from January 2012 to December 2017. Rate of CRRT application, along with patients' clinical data at PICU admission [i.e., anthropometric and clinical characteristics, such as admission diagnosis, urinary output (UO), accumulated FO, need for vasopressors or mechanical ventilation] are described. In the subgroup of patients where data on CRRT prescription and delivery were available, we also described patients' clinical characteristics at CRRT initiation and CRRT technical features. In particular, UO, FO, and laboratory data were collected immediately before CRRT initiation, along with timing-to-start and indication for CRRT. Being a retrospective observational study, CRRT initiation was determined by the attending physicians according to local policy and practice. Technical data include information on CRRT initial prescription (i.e., treatment modality, filter type, treatment duration, adopted vascular access, anticoagulation strategy), and minute-by-minute treatment delivery information recorded on the CRRT monitor memory card (e.g., flows, pressures, and machine alarms). Combined analysis of overtime pressure drop and overtime transmembrane pressure (TMP) and machine alarms allowed the identification of unintended discontinuation sessions due to membrane fouling. Stopped sessions were reported as “clotted” if pressure drop had increased above 150 mmHg and/or clotting machine alarms had been identified in the machine recorded treatment data. Technical CRRT data regarding CRRT prescription, available for each delivered session, were used to identify predictors for circuit clotting. Recorded treatment data were analyzed to identify treatment clogging (TMP elevation >250 mmHg) and vascular access dysfunction (negative access pressure, below −100 mmHg for a cumulative time longer than 120 min). Patients experiencing a rate of clotted sessions >25% of total CRRT sessions were considered as patients with “high clotting rate.” On this basis, patients' clinical and laboratory characteristics were also reported according to clotting rate.

Finally, mortality rate at PICU discharge, long-term patient survival, proteinuria or chronic kidney disease (CKD) development at last available follow-up, and referral to nephrology outpatient clinic were evaluated. Patients were considered lost to follow-up if data in the hospital electronic chart were not available. Three authors (EB, BT, and FG) independently performed data extraction and collection and subsequently cross-checked the results. Discrepancies were re-examined by GV. AKI was defined according to KDIGO criteria (19), while percentage of FO was defined as [(fluid in – fluid out)/PICU admission weight]^*^100, as previously described (20). Proteinuria and CKD were defined based on KDIGO definitions (urinary albumin-to-creatinine ratio >3 mg/mmol; eGFR <90 ml/min/1.73 m^2^, bed-side Schwartz formula), while end stage kidney disease (ESKD) was defined by initiation of chronic replacement therapy (intermittent hemodialysis or kidney transplantation) (21). Patients' clinical and laboratory characteristics were also reported according to PICU survival.

### Statistical Analysis

Descriptive statistics were reported as appropriate after testing continuous variables for normality of the distribution by the Shapiro-Wilk test. Frequency and percentage were reported for qualitative variables, while mean and standard deviation were calculated for quantitative variables. Median and interquartile range (IQR) were calculated for quantitative variables with non-normal distribution. Kaplan-Meier survival analysis was run to evaluate parameters associated with premature clotting. Univariate logistic regression and Cox regression analysis were performed to estimate the size of association between clinical and technical variables and patient's clotting rate, circuit premature clotting, and PICU survival. Odds ratio (OR), hazard ratio (HR) and their 95% confidence interval (95%CI) were reported. For circuit clotting prediction, variables with a Wald test's *p*-value < 0.10 in the univariate analysis were considered for multivariate Cox regression analysis. Independent predictors for premature clotting were identified through backward selection based on the AIC. Statistical significance was set to *p*-value < 0.05. Statistical analysis was performed using R^©^ software version 3.5.1.

### Ethical Concerns

The present study has been approved by the Meyer Children's Hospital Ethics Committee (registry number 104/2020). Being an observational study, the Ethics Committee waived informed consent for the analysis. Patients enrolled in this study did not receive additional medical, pharmacological or behavioral interventions other than those routinely administered in the PICU. Research was carried out in agreement with the principles of the original Declaration of Helsinki and its later amendments.

## Results

### Rate of CRRT Application and Patients' Characteristics at PICU Admission

Of the 1,996 patients admitted to the Meyer Children's Hospital PICU in the 6-year study period, 28 patients (1.4%) received CRRT (Table 1). In this cohort of CRRT patients, median age at PICU admission was 2 years [1–6], with a slightly higher proportion of females (15/28, 53.6%). Median height, weight, and body surface area were 90 cm [75–108], 13.0 Kg [9.8–21.3], and 0.58 m^2^ [0.45–0.84], respectively. PICU admission diagnoses were: respiratory failure, pneumonia or respiratory distress (10/28, 35.7%), septic shock (5/28, 17.9%), onco-hematological disease (5/28, 17.9%), hemolytic uremic syndrome, severe AKI or rhabdomyolysis (5/28, 17.9%), and others (3/28, 10.7%, macrophage activation syndrome *n* = 1, pulmonary hypertension *n* = 1, X-linked chronic granulomatosis *n* = 1) (Table 1). At PICU admission, median urinary output and fluid overload were 2.00 ml/Kg/h [0.36–3.82] and 3.2% [0.6–5.5], respectively, while mechanical ventilation was needed in 24/28 (85.7%) patients. Hemodynamic instability requiring vasopressors was observed in 14/28 (50%) cases at PICU admission (Table 1).

**Table 1:** Demographic and clinical characteristics at pediatric intensive care unit admission.

**ID**	**Sex, age**	**Height (cm), Weight (Kg)**	**BSA (m^**2**^)**	**Admission diagnosis**	**UO (ml/Kg/h), FO (%)**	**VP—MV**
1	M, 1d	56, 3	0.21	Meconium aspiration	4.0, 22.0	1–1
2	M, 8d	60, 3	0.22	Pulmonary HTN	2.7, 0.5	1–1
3	M, 2m	52, 4	0.23	TAPVR	2.5, −0.5	1–1
4	M, 2m	60, 6.6	0.31	Septic shock	0.0, 0.0	1–1
5	M, 5m	62, 6	0.31	Tracheomalacia	4.2, 1.7	0–0
6	M, 11m	75, 9	0.42	HUS	0.4, 8.0	0–1
7	M, 1y	52, 2	0.17	Pneumonia	8.6, 1.5	1–1
8	F, 1y	75, 10	0.44	Severe AKI	0.1, 3.9	0–1
9	M, 1y	82, 10	0.47	T cell leukemia	0.3, 7.5	1–1
10	F, 1y	82, 12	0.50	HUS	0.7, 5.7	0–1
11	F, 2y	81, 12	0.50	Pneumonia	3.8, 6.2	1–1
12	F, 2y	90, 12	0.54	Pneumonia	0.4, 10.4	0–0
13	F, 2y	100, 12	0.58	Septic shock	8.2, 3.1	0–1
14	F, 2 y	91, 14	0.58	MAS	0.6, 4.0	1–1
15	M, 2y	92, 14	0.59	T cell leukemia	3.4, 2.8	0–1
16	M, 4y	90, 13	0.56	Pneumonia	3.3, 4.2	0–1
17	F, 4y	92, 16	0.62	Severe AKI	3.7, −2.7	0–1
18	M, 4y	98, 14	0.61	X-CGD	5.9, 1.1	1–1
19	F, 5y	96, 13	0.58	Pulmonary edema	0.7, 3.5	0–1
20	F, 5y	86, 17	0.61	FB ingestion	0.0, 3.3	1–1
21	F, 5y	105, 25	0.82	Pneumonia	0.2, 0.7	0–1
22	F, 9y	120, 29	0.97	B cell leukemia	1.5, 0.9	1–0
23	F, 10y	160, 75	1.78	Rhabdomyolysis	6.5, −1.5	1–1
24	F, 11y	125, 20	0.85	Septic shock	0.0, 5.4	1–1
25	M, 12y	130, 26	0.98	Septic shock	0.2, −2.8	0–1
26	M, 15y	163, 68	1.73	B cell leukemia	1.1, 0.1	0–1
27	F, 16y	165, 52	1.56	ALL	3.5, 3.7	1–1
28	F, 17y	168, 50	1.56	Septic shock	5.5, 7.3	0–0

*Age (d, days; m, months; y, years); AKI, acute kidney injury; ALL, acute lymphoblastic leukemia; BSA, body surface area; FB, foreign body; FO, fluid overload; HTN, hypertension; HUS, hemolytic uremic syndrome; MAS, macrophage activation syndrome; MV, need for mechanical ventilation; PICU, pediatric intensive care unit; Sex (F, female; M, male); TAPVR, total anomalous pulmonary venous return; UO, urinary output; VP, need for vasopressors; X-CGD, x-linked chronic granulomatous disease; 0 = no; 1 = yes*.

### CRRT Indication and Prescription

Clinical characteristics of 23 patients are described in Table 2. All of the 23 patients were treated with Prismaflex® (Baxter, Deerfield, Illinois) machine, for a total of 101 treatment sessions.

**Table 2:** Clinical and biochemical characteristics at continuous renal replacement therapy initiation; number, duration, and clotting rate.

**ID**	**Time-to-start (h)**	**Indication**	**UO (ml/Kg/h), FO (%)**	**sCr (umol/L)**	**BUN (mmol/L)**	**K (mmol/L)**	**CRRT sessions**	
							**Number, mean duration (h)**	**Clotting rate**
1	17	FO	1.8, 24.1	92.8	3.2	5.9	10, 27.5	2/10
2	150	FO	1.1, 31.1	61.9	22.8	3.5	3, 21.1	3/3
5	217	FO	1.3, 120.7	35.4	6.0	3.8	5, 29.3	3/5
6	7	Hyperazotemia	0.1, 0.4	594.2	51.2	4.8	5, 38.4	1/5
7	395	FO	4.0, 200.0	77.8	37.3	6.6	4, 12.3	1/4
8	4	Hyperazotemia	0.0, 1.8	634.0	58.2	6.5	9, 20.1	8/9
9	41	FO	0.5, 27.4	114.9	29.3	4.7	4, 16.4	4/4
10	60	FO	0.2, 21.1	201.6	20.3	3.4	2, 21.5	2/2
11	74	FO	5.1, 12.4	62.8	13.0	3.5	1, 22.2	1/1
12	18	FO	0.5, 15.1	142.4	19.5	3.6	5, 49.7	4/5
13	27	FO	2.1, 10.4	114.9	21.7	4.3	7, 10.0	6/7
14	28	FO	1.5, 8.2	22.1	8.0	5.1	3, 48.9	0/3
15	168	FO	2.1, 18.5	82.2	22.5	4.6	6, 66.5	2/6
16	219	Hyperkalemia	0.3,−4.1	133.5	37.3	6.0	7, 74.9	0/7
18	40	FO	1.8, 10.8	79.6	7.3	3.4	4, 59.7	2/4
19	11	Hyperazotemia	0.4, 4.7	847.1	44.3	4.1	1, 9.8	0/1
20	22	Hyperkalemia	0.0, 2.8	132.6	6.0	5.9	1, 4.5	1/1
21	7	Hyperazotemia	0.1, 0.7	610.1	48.3	4.5	3, 68.8	0/3
22	188	FO	0.1, 8.9	70.7	6.7	3.3	1, 32.1	1/1
23	61	Hyperazotemia	0.8, 4.9	448.3	29.3	4.8	8, 49.5	7/8
25	29	Shock in IHD	1.9, 0.1	IHD	20.3	5.4	6, 31.0	3/6
26	38	Hyperazotemia	0.7,−0.2	154.7	44.8	4.4	4, 64.2	2/4
28	210	FO	2.7, 28.6	238.7	24.5	4.5	2, 79.8	0/2

*Clotting rate represents the proportion of clotted sessions for each patient. A clotting rate > than 25% identifies patients at “high clotting rate.” BUN, blood urea nitrogen; FO, fluid overload; IHD, intermittent hemodialysis; K, potassium; sCr, serum creatinine; Time-to-start, time from admission to CRRT initiation; UO, urinary output*.

Median time from PICU admission to CRRT initiation was 40 h [20–160], and indications for CRRT initiation were fluid overload in 14/23 (60.9%) patients, hyperazotemia and/or hyperkalemia in 8/23 (34.8%) patients. At CRRT initiation, median UO, FO, serum creatinine, blood urea nitrogen, and potassium were 0.75 ml/Kg/h [0.27–1.89], 10.4% [2.3–22.6], 124 umol/L [78–229] (1.4 mg/dl [0.9–2.6]), 22.5 mmol/L [10.5–37.3] (63 mg/dl [29–105]), and 4.5 mmol/L [3.7–5.3], respectively (Table 2). Median hemoglobin, hematocrit, and calcium levels were 6.2 mmol/L [5.3–7.1], 28.9% [25.7–34.6], and 2.26 mmol/L [1.96–2.44], respectively. Table 2 also shows the number of CRRT sessions for each treatment, as well as mean session duration, and proportion of clotted sessions for each patient. Clotting rate was higher than 25% in 15 patients (65.2%).

Among the 101 CRRT sessions, the most common prescription was Continuous Veno-Venous Hemodiafiltration (CVVHDF) (84/101, 83.2%), and 3/4 treatments were performed with a AN69ST membrane (acrylonitrile and sodium methallyl sulfonate copolymer) (Table 3). Filters with surface area smaller than 0.6 m^2^ were used in 13.8% of sessions. The most frequently used access site was the femoral vein, and 17 sessions were linked to an ECMO circuit (patient IDs 1, 2, 11, 18). Median access length and caliber were 12 cm and 8.0 Fr, respectively. In all patients, packed red blood cells were used to prime the extracorporeal circuit. The most common anticoagulation method was continuous systemic unfractioned heparin (72/101, 71.3%) with an average dose of 13.9 U/Kg/h, while regional citrate anticoagulation (RCA) was used in 11/101 (10.9%) sessions. Eighteen sessions (17.8%) were performed with no anticoagulation for clinical decision. CRRT was continued for a median of 12 days [7.75–17] and sessions lasted for a median of 30.2 h [7.1–65.6].

**Table 3:** Prescription characteristics and clotting predictors of continuous renal replacement therapy sessions.

**Parameter**	**Total (*n* = 101)**	**No clotting (*n* = 42)**	**Clotting (*n* = 59)**	**HR [95% CI]**	***P***
**Treatment modality**					0.059
CVVHD	16 (15.8%)	3 (18.8%)	13 (81.2%)	Ref.	
CVVHDF	84 (83.2%)	39 (47.0%)	45 (53.0%)	0.50 [0.27–0.94]	**0.031**
SCUF	1 (1.0%)	0 (0.0%)	1 (100.0%)	1.64 [0.21–12.76]	0.635
**Filter type**					**0.033**
HF1000	4 (4.0%)	0 (0.0%)	4 (100.0%)	Ref.	
HF20	14 (13.9%)	4 (28.6%)	10 (71.4%)	1.47 [0.46–4.71]	0.520
M60	16 (15.8%)	8 (50.0%)	8 (50.0%)	0.53 [0.16–1.78]	0.304
ST60	39 (38.6%)	16 (41.0%)	23 (59.0%)	0.65 [0.22–1.91]	0.437
ST100	15 (14.9%)	10 (66.7%)	5 (33.3%)	0.28 [0.07–1.07]	0.063
ST150	6 (5.9%)	3 (50.0%)	3 (50.0%)	0.35 [0.07–1.56]	0.168
SEPTEX	7 (6.9%)	1 (14.3%)	6 (85.7%)	1.26 [0.36–4.49]	0.718
**Membrane type**					**0.005**
PAES	18 (17.8%)	4 (22.2%)	14 (77.8%)	Ref.	
AN69ST	76 (75.2%)	37 (48.7%)	39 (51.3%)	0.39 [0.21–0.74]	**0.004**
PAES-HCO	7 (6.9%)	1 (14.3%)	6 (85.7%)	0.98 [0.38–2.56]	0.969
**Filter area**					**0.030**
0.2 m^2^	14 (13.8%)	4 (28.6%)	10 (71.4%)	Ref.	
0.6 m^2^	55 (54.5%)	24 (43.6%)	31 (56.4%)	0.43 [0.21–0.88]	**0.022**
≥ 1 m^2^	32 (31.7%)	14 (43.6%)	18 (56.4%)	0.36 [0.16–0.79]	**0.010**
**Prescription flows**					
Blood flow (ml/min)	60 [40–80]	60 [50–100]	60 [40–80]	0.99 [0.99–1.00]	0.379
Dialysate flow (ml/h)	400 [200–600]	400 [250–500]	400 [100–800]	1.00 [1.00–1.00]	0.073
Replacement flow (ml/h)	200 [50–400]	200 [50–500]	200 [0–400]	0.99 [0.99–1.00]	0.309
Net ultrafiltration (ml /h)	40 [25–70]	40 [25–60]	40 [25–70]	1.00 [0.99–1.00]	0.726
Effluent flow (ml /h)	900 [510–1120]	790 [525–1080]	910 [390–1130]	1.00 [0.99–1.00]	0.739
Filtration fraction (%)	17.1 [9.1–23.8]	15.6 [5.7–22.4]	18.7 [11.3–23.9]	1.00 [0.98–1.03]	0.849
**Vascular access site**					0.175
Jugular	29 (28.7%)	18 (62.1%)	11 (37.9%)	Ref.	
Femoral	55 (54.5%)	20 (36.4%)	35 (63.6%)	1.51 [0.76–2.97]	0.238
ECMO	17 (16.8%)	4 (23.5%)	13 (76.5%)	2.16 [0.96–4.82]	0.062
**Vascular access caliber**	8.0 [8.0–8.5]				**0.004**
≤ 8 Fr	65/92 (70.7%)	24 (36.9%)	41 (63.1%)	Ref.	
> 8 Fr	27/92 (29.3%)	16 (59.3%)	11 (40.7%)	0.37 [0.19–0.72]	
**Vascular access length**	12.0 [11.5–14.0]				0.806
≤ 12 cm	46/71 (64.8%)	22 (47.8%)	24 (52.2%)	Ref.	
> 12 cm	25/71 (35.2%)	10 (40.0%)	15 (60.0%)	0.92 [0.48–1.76]	
**Anticoagulation**					**0.010**
None	18 (17.8%)	5 (27.8%)	13 (72.2%)	Ref.	
Heparin	72 (71.3%)	28 (39.4%)	44 (60.6%)	0.75 [0.40–1.40]	0.376
Citrate	11 (10.9%)	9 (81.8%)	2 (18.2%)	0.13 [0.03–0.59]	**0.008**

*AN69ST, acrylonitrile and sodium methallyl sulfonate copolymer; CVVHD(F), continuous veno-venous hemodialysis (Hemodiafiltration); HCO, high cut-off; HR, hazard ratio; PAES, polyarylethersulfone; Ref., reference; SCUF, slow continuous ultrafiltration*.

### Predictors of Unintended Discontinuation Due to Clotting

**Figure 1:**
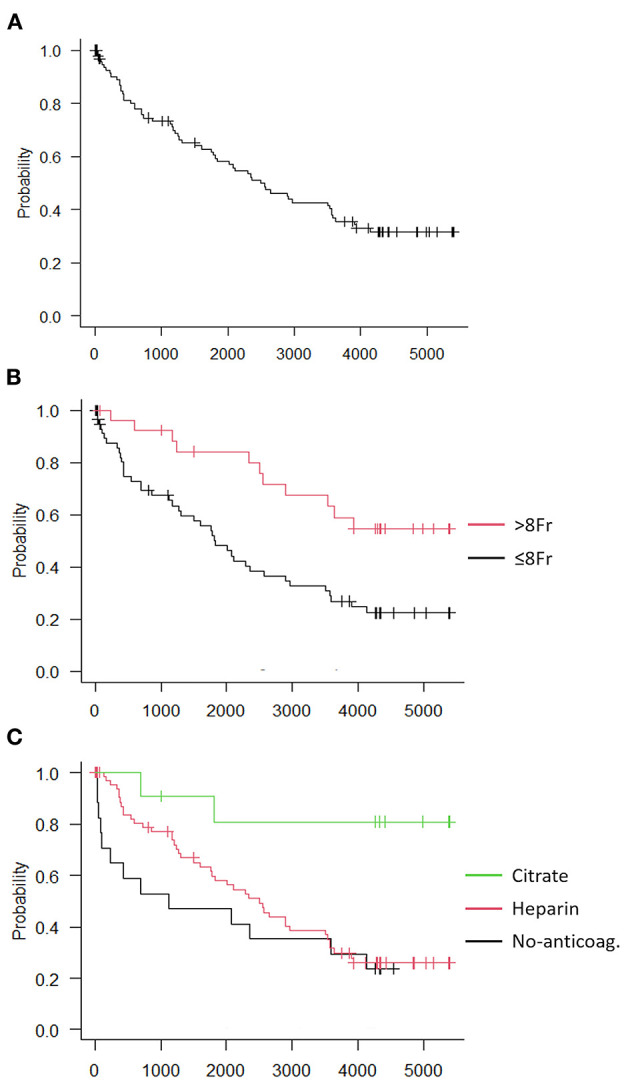
Kaplan-Meier curves of session-related clotting independent risk factors. **(A)** Clotting-free survival analysis of all 101 sessions, **(B)** Sessions divided according to vascular access caliber, **(C)** Sessions divided according to anticoagulation method.

Unintended treatment discontinuation due to clotting was detected in more than half of CRRT sessions (59/101, 58.4%), after a median session duration of 21.2 h [7.1–42.1]. Every clotted session showed an increase in TMP (indicating membrane clogging in most of the cases), and 50/59 (84.7%) sessions also presented signs of vascular access dysfunction. Variables significantly associated with clotting in the univariate Cox regression analysis were membrane type (*p* = 0.005), filter area (*p* = 0.03), vascular access caliber (*p* = 0.004), and anticoagulation strategy (*p* = 0.01) (Table 3). In particular, most of the sessions performed with a polyarylethysulfone (PAES) high-flux membrane (77.8%), using a filter with smaller surface area (71.4%), via a smaller vascular access (≤ 8 Fr, 63.1%), and without regional citrate anticoagulation (72.2 and 60.6%, respectively, for no-anticoagulation and systemic heparinization) underwent premature clotting. Initial prescription flows were not significantly different between clotting and no-clotting groups. Multivariate Cox regression analysis identified dimension (in Fr) of the vascular access [HR 0.37 (0.19–0.72), *p* = 0.004] and RCA strategy [HR 0.14 (0.03–0.60), *p* = 0.008] as two baseline independent predictors for premature clotting (Figure 1). Among the 23 patients, 8 (34.8%) experienced clotting in ≤25% of CRRT sessions and were thus considered as patients with “low clotting rate.” On the other hand, the remaining 15 (65.2%) patients had clotting in more than 25% of CRRT sessions and were thus considered as patients with “high clotting rate.” Patients' clinical characteristics at CRRT initiation are described for both groups in Supplementary Table 1. No significant clinical predictors were found in these groups.

### Short- and Long-Term Outcomes

Considering the whole study population (*n* = 28), average PICU length of stay was 24 days [12–30], with a PICU mortality rate of 42.8% (12/28). Baseline clinical and CRRT characteristics of patients who died and patients who survived to PICU discharge are described in Supplementary Table 2. Requirement of vasoactive treatment was the only predictor of mortality among these subgroups. Of the 16/28 CRRT patients successfully discharged from the PICU, one was a ESKD patient already treated with IHD before PICU admission; three were lost to follow-up soon after discharge; the remaining 12 had at least 1 year follow-up, with a mean follow-up length of 3.5 ± 2.0 years. Among these 12 patients, only 5 (42%) did not develop any form of kidney dysfunction, one (8%) developed low grade proteinuria, three (25%) developed CKD, and three (25%) developed ESKD (Table 4). Of note, 3/5 (60%) patients who did not develop kidney disease have a follow-up of only 1 year after discharge. Overall, 7/15 patients (46.7%) were never referred to nephrology follow-up after PICU discharge.

**Table 4:** Long-term follow-up after pediatric intensive care unit survival.

**ID**	**PICU stay (d)**	**PICU survival**	**Follow-up data**	**Follow-up time (y)**	**Kidney outcome**
1	30	yes	yes	6.5	ok
6	22	yes	yes	3	proteinuria
8	27	yes	yes	6	ESKD
10	13	yes	yes	1	ok
11	25	yes	yes	1	ok
12	30	yes	yes	3.5	CKD
13	29	yes	no	-	-
14	25	yes	no	-	-
16	97	yes	yes	1	CKD
17	4	yes	yes	6	CKD
19	26	yes	yes	5	ESKD
21	40	yes	no	-	-
23	34	yes	yes	2.5	ok
24	51	yes	yes	6	ESKD
25	12	yes	IHD		
28	52	yes	yes	1	ok

*CKD, chronic kidney disease; ESKD, end-stage kidney disease; IHD, intermittent hemodialysis; PICU, pediatric intensive care unit; Time (d, days; y, years)*.

## Discussion

In this single center retrospective study, we have observed a rate of CRRT application of 1.4% in a cohort of pediatric critically ill patients admitted to a tertiary pediatric hospital. In our cohort, extracorporeal treatments were applied within 48 h after PICU admission and mainly for management of fluid overload. Most of the treatments were performed in CVVHDF modality, with large (>0.6 m^2^) acrylonitrile high-flux membranes and systemic heparinization. Large vascular access (>8 Fr) and RCA were independent protective factors for circuit clotting. Patients' PICU mortality rate was high and survival often complicated by CKD development.

In the context of a general lack of clinical and technical information on CRRT in PICU, here we accurately describe our cohort of pediatric CRRT patients in terms of clinical presentation, treatment prescription, and CRRT indication. Our results are in agreement with the available literature. The findings confirm the relative low rate of application of CRRT in PICU and describe FO as the main indication for CRRT initiation (22). In the literature, the most frequent clinical indication for CRRT is severe AKI complicated with the concomitant the requirement of fluid administration (diuretic-unresponsive oligo-anuria and subsequent FO) and/or metabolic (untreatable acidosis, hyperkalemia, and uremic toxins accumulation) disturbances (23). However, no clear cut-off values for CRRT initiation are currently available and, as a consequence, timing is controversial, even in adult patients (24–29). Since almost two decades, FO has been identified as a main independent predictor of mortality in the PICU setting (14, 20). The US multicenter, prospective, pediatric CRRT (ppCRRT) registry has led to numerous studies addressing diverse clinical questions about CRRT patients and modalities (6). Evidence from these studies suggests that survival is greatly influenced by the underlying disease at admission. It also highlights the importance of circuit survival and nutritional prescription, and it confirms the independent association between FO at CRRT initiation and mortality (15, 30). However, these studies failed to define a target %FO for CRRT initiation or to determine if aggressive treatment of FO could improve survival in these patients (6). It has been suggested that CRRT should be started rapidly in oligo-anuric patients, before a FO threshold of 10–20% is reached (15). Retrospective cohort studies suggest that earlier CRRT initiation is associated with improved survival (31), with mortality increasing per each hour of delay (16), but other studies also stress that CRRT initiation confers a more than eight-fold higher mortality risk with respect to the total PICU population (17). In fact, CRRT patients, especially children, can experience complications related to vascular access placement, anticoagulation and blood loss, hypotension, and electrolytes derangement (32–34). Particularly in small pediatric patients, maintenance of filter patency and avoidance of premature clotting are crucial to increase safety, efficacy and effectiveness of the treatment (9). Beside undertreatment caused by membrane fouling and downtime due to circuit substitution (35), the amount of blood retained into the extracorporeal circuit for unexpected clotting can be clinically relevant in a small pediatric patient. Risk factors for filter clotting should be explored in pediatric population and clinical practice improved in order to minimize this harmful complication.

In line with previous experiences reported in the literature (36, 37), we found that dimension of vascular access and anticoagulation strategy were independent predictors for circuit clotting. In particular, smaller vascular access (≤ 8 Fr) was significantly associated with filter clotting. This was likely associated with the fact that treatments in smaller patients, carrying the smallest catheters sizes were those complicated by more frequent unintended interruptions. Interestingly, overtime analysis of access pressure during treatment revealed signs of vascular access dysfunction in most of the clotted sessions. Thus, when possible, a larger catheter should always be used for vascular access. Unfortunately, use of large catheters is often not feasible in newborn or small pediatric patients. In these cases, the adoption of hardware components specifically designed for pediatric patients might help delivering an adequate CRRT session. It is also possible that the internal jugular vein might be preferred in order to optimize circuit patency (6). As described in our population, however, this access in critically ill children may frequently be already utilized for a central venous catheter and the only available option could be to select the femoral vein. Even if in our study access site did not show significant differences in terms of clotting rate, it might be interesting in larger studies to further address this important aspect. Again, regardless of vascular access site and size, miniaturized disposable, filters, and roller pumps, mainly, might reduce the amplitude of excessive cyclic pressure oscillations, led by large peristaltic pumps flowing fluids against small tubes and vascular accesses (38, 39). In this context, Carpediem® (Cardio Renal Pediatric Emergency Machine) (Bellco-Medtronic, Mirandola, Italy) might be proposed for smaller patients, especially those with “high clotting rate” characteristics. Interestingly, despite the relatively small size of patients enrolled in our cohort, filters with surface area smaller than 0.6 m^2^ were used in <15% of sessions. Moreover, our results confirm RCA as a major independent protective factor to reduce circuit clotting, compared to heparin or no-anticoagulation strategies. Therefore, also according to the literature, RCA should be adopted as a first choice anticoagulation strategy for CRRT.

Mortality in our study was similar to that reported in the ppCRRT study (42%) (6) but higher than the results of the AWARE trial (25%) (2). The latter study involved 4,984 critically ill children and young adults and described RRT as one of the most important predictors of mortality in the PICU [OR 3.38 (1.74–6.54)] (2). Moreover, it is possible that the population described in our study was mainly composed by children with multiple organ failure, similarly to that reported in the pediatric registry (6). The availability of data on long-term renal function is the last crucial finding of this study. According to our data, the majority of children undergoing CRRT developed some form of chronic kidney dysfunction. Few studies are available on CKD following pediatric AKI and their results appear controversial, with the rate of uncomplete recovery of renal function ranging from 10 to 20% (16, 40). This aspect likely depends on the severity of AKI of analyzed patients (16), their age (41), admission diagnosis (42), and effectiveness of follow-up (43). Interestingly, about 60% of our cohort underwent a post PICU discharge renal referral and follow-up which is higher than recently described (44). This was probably due to the identification of the high severity of renal dysfunction in patients who required dialysis during their PICU admission. In this regard, it would be desirable that close to 100% of AKI children requiring CRRT were referred to the nephrology consultation and follow-up. Unfortunately, this target is far to be reached in clinical practice and very limited information is available in the literature on long-term outcomes of pediatric patients treated with CRRT (45).

### Limitations

Several drawbacks should be recognized in this study. Although the relatively small sample size is a major limitation, the single center nature of our study has allowed to accurately describe each patient's clinical presentation at admission and at CRRT initiation, treatment prescription, characteristics, and each circuit lifespan, and also to describe short- and long-term survival and kidney outcome in a precise setting. Unfortunately, we were not able to present data for the entire population of 28 patients who underwent CRRT, but only for the subgroup of 23 patients treated with Prismaflex® machine. Multicenter registries (46) are certainly required for the observation of larger populations. Duration of follow-up was relatively short, and it is possible that longer observation may reveal different outcomes: it is currently unknown if these would imply improvement of further worsening of renal function of these patients. Given the observation nature of this study it is currently unknown if timing of CRRT start, severity of the admission disease, and dose of the analyzed treatment could have affected short and long-term outcomes. However, literature in this field of pediatric critical care nephrology is poor and consistent results should be extrapolated by large databases.

## Conclusions

Our data indicate a low prevalence of CRRT in the PICU population and confirm high morbidity and mortality in these patients. Pediatric CRRT administration is often complicated by unintended discontinuation due to circuit clotting and loss of effective treatment time that should be taken into account early during treatment prescription. Use of adequate vascular accesses and RCA might protect the circuit from clotting. Moreover, despite a relatively short median follow-up time, a great proportion of CRRT patients developed CKD and needed nephrology consult. Once more, this highlights the importance of nephrology referral for these patients, from first CRRT prescription to, more importantly, post-discharge outpatient care.

## Data Availability Statement

The original contributions generated for the study are included in the article/Supplementary Material, further inquiries can be directed to the corresponding author/s.

## Ethics Statement

The studies involving human participants were reviewed and approved by the Meyer Children's Hospital Ethics Committee (registry number 104/2020). Research was performed in line with the principles of the Declaration of Helsinki. Written informed consent for participation was not provided by the participants' legal guardians/next of kin because the Ethics Committee waived informed consent for the analysis and publication.

## Author Contributions

EB and FG contributed equally to the work, conceived the research idea and the design of the study, were responsible for material preparation and data collection and wrote the first draft of the manuscript. DC and BT contributed to material preparation and data collection. GV was responsible for statistical analysis. SR, ZR, ML'E, and GV contributed to the conception and design of the study. All authors commented on previous versions of the manuscript, read and approved the final manuscript providing substantial contribution as per ICMJE recommendations.

## Conflict of Interest

GV has received honoraria for lectures from Baxter and Pall Italia. SR has received honoraria for lectures/consultancy from Baxter, Orion Pharma, Vygon, MSD, and Medtronic and funds for travel expenses, hotel accommodation and registration to meetings from Baxter, BBraun, Pall International, Medigas and Vygon. The remaining authors declare that the research was conducted in the absence of any commercial or financial relationships that could be construed as a potential conflict of interest.
