# Supplementary material for: Continuous Renal Replacement Therapy in Critically Ill Children in the Pediatric Intensive Care Unit: A Retrospective Analysis of Real-Life Prescriptions, Complications, and Outcomes
Source: Front Pediatr. 2021 Jun 14;9:696798. doi: 10.3389/fped.2021.696798 (PMC8236631; doi:10.3389/fped.2021.696798)
Supplement: Supplementary file 1 [file Data_Sheet_1.PDF]

Supplementary Material

Supplementary Table 1 – Clinical characteristics according to patient's clotting rate

Characteristics	Total (n=23)	Low clotting rate (n=8)	High clotting rate (n=15)	OR [95%CI]	*p*
Age (y)	2 [1-5]	3 [1-5]	2 [1-9]	1.00 [0.84-1.20]	0.986
Gender (female)	12 (52%)	4 (33%)	8 (67%)	0.88 [0.16-4.87]	0.879
Height (cm)	90 [75-105]	90 [65-100]	90 [81-120]	1.01 [0.98-1.04]	0.612
Weight (kg)	13 [10-25]	13 [6-20]	12 [10-26]	1.02 [0.97-1.07]	0.539
BSA (m^2)	0.58 [0.44-0.82]	0.57 [0.32-0.70]	0.58 [0.44-0.82]	1.84 [0.22-15.70]	0.576
Vasopressors need (y)	10 (44%)	3 (30%)	7 (70%)	1.46 [0.25-8.43]	0.673
Mechanical ventilation	19 (83%)	7 (37%)	12 (63%)	0.57 [0.05-6.61]	0.654
Adm-to-CRRT (h)	40 [18-168]	22 [9-214]	41 [27-150]	0.99 [0.98-1.01]	0.415
K-pre (mmol/L)	4.5 [3.6-5.4]	4.8 [4.5-5.9]	4.3 [3.5-4.8]	0.40 [0.15-1.08]	0.070
Hct-pre (%)	28.9 [25.4-35.6]	27.9 [26.2-34.8]	29.0 [24.4-35.6]	0.97 [0.87-1.08]	0.588
BUN-pre (mmol/L)	22.5 [7.8-37.5]	37.1 [16.1-46.4]	20.4 [7.1-29.3]	0.99 [0.97-1.01]	0.186
Fluid overload (%)	10.3 [1.8-24.1]	6.4 [0.5-26.4]	10.8 [2.8-21.1]	0.99 [0.98-1.01]	0.500
CRRT length (d)	10 [6-17]	11 [10-20]	8 [3-14]	0.92 [0.81-1.04]	0.171
Sessions (n)	4 [2-6]	4 [3-6]	4 [2-6]	1.00 [0.72-1.41]	0.982

Demographic, anthropometric, clinical, and laboratory characteristics of the 23 patients are shown together with the results of a univariate logistic regression analysis according to patient's clotting rate. A >25% rate of clotted sessions was used as threshold to define patients at "high clotting rate".

Supplementary Table 2 – Clinical characteristics according to Pediatric Intensive Care Unit survival

Characteristics	Total (n=23)	PICU survival (n=14)	PICU death (n=9)	HR [95%CI]	*p*
Age (y)	2 [1-5]	2 [1-5]	2 [1-5]	1.01 [0.88-1.16]	0.903
Gender (female)	12 (52%)	10 (83%)	2 (17%)	4.04 [0.81-20.26]	0.089
Height (cm)	90 [75-105]	90 [81-105]	86 [62-98]	0.99 [0.97-1.02]	0.750
Weight (kg)	13 [10-25]	12 [12-25]	14 [6-17]	0.99 [0.96-1.03]	0.892
BSA (m^2)	0.58 [0.44-0.82]	0.57 [0.50-0.82]	0.58 [0.31-0.61]	0.83 [0.17-4.05]	0.820
Vasopressors need (y)	10 (43%)	4 (40%)	6 (60%)	5.63 [1.13-28.08]	**0.035**
Mechanical ventilation	19 (83%)	12 (63%)	7 (37%)	1.68 [0.21-13.66]	0.628
Adm-to-CRRT (h)	40 [18-168]	28 [11-61]	150 [40-188]	1.00 [0.99-1.01]	0.238
K-pre (mmol/L)	4.5 [3.6-5.4]	4.5 [4.1-5.4]	4.4 [3.5-4.7]	0.80 [0.42-1.87]	0.749
Hct-pre (%)	28.9 [25.4-35.6]	26.6 [24.2-28.9]	32.5 [30.1-36]	1.04 [0.97-1.11]	0.265
BUN-pre (mmol/L)	22.5 [7.8-37.5]	23.2 [19.6-44.3]	22.5 [6.8-29.3]	0.99 [0.98-1.01]	0.420
Fluid overload (%)	10.3 [1.8-24.1]	6.5 [0.7-15.0]	18.5 [8.9-31.1]	0.99 [0.99-1.00]	0.681
CRRT length (d)	10 [6-17]	11 [8-18]	7 [3-10]	0.89 [0.79-1.01]	0.087
Sessions (n)	4 [2-6]	5 [2-7]	4 [3-4]	0.79 [0.58-1.08]	0.145
Clotted sessions (%)	50 [20-94]	35 [0-88]	60 [50-100]	1.02 [0.99-1.04]	0.108

Demographic, anthropometric, clinical, and laboratory characteristics of the 23 patients are shown together with results of univariate Cox regression analysis according to PICU survival.
